# Diosgenin: An Updated Pharmacological Review and Therapeutic Perspectives

**DOI:** 10.1155/2022/1035441

**Published:** 2022-05-29

**Authors:** Prabhakar Semwal, Sakshi Painuli, Tareq Abu-Izneid, Abdur Rauf, Anshu Sharma, Sevgi Durna Daştan, Manoj Kumar, Mohammed M. Alshehri, Yasaman Taheri, Rajib Das, Saikat Mitra, Talha Bin Emran, Javad Sharifi-Rad, Daniela Calina, William C. Cho

**Affiliations:** ^1^Department of Biotechnology, Graphic Era University, Dehradun, 248002 Uttarakhand, India; ^2^Pharmaceutical Sciences Department, College of Pharmacy, Al Ain University, Al Ain 64141, UAE; ^3^Department of Chemistry, University of Swabi, Swabi, Anbar-23561, K.P .K, Pakistan; ^4^Department of Food Science and Technology, Dr. Y.S. Parmar University of Horticulture and Forestry, Nauni, 173230, India; ^5^Department of Biology, Faculty of Science, Sivas Cumhuriyet University, 58140 Sivas, Turkey; ^6^Beekeeping Development Application and Research Center, Sivas Cumhuriyet University, 58140 Sivas, Turkey; ^7^Chemical and Biochemical Processing Division, ICAR-Central Institute for Research on Cotton Technology, Mumbai 400019, India; ^8^Pharmaceutical Care Department, Ministry of National Guard-Health Affairs, Riyadh, Saudi Arabia; ^9^Phytochemistry Research Center, Shahid Beheshti University of Medical Sciences, Tehran, Iran; ^10^Department of Pharmacy, Faculty of Pharmacy, University of Dhaka, Dhaka 1000, Bangladesh; ^11^Department of Pharmacy, BGC Trust University Bangladesh, Chittagong 4381, Bangladesh; ^12^Department of Pharmacy, Faculty of Allied Health Sciences, Daffodil International University, Dhaka 1207, Bangladesh; ^13^Facultad de Medicina, Universidad del Azuay, Cuenca, Ecuador; ^14^Department of Clinical Pharmacy, University of Medicine and Pharmacy of Craiova, 200349 Craiova, Romania; ^15^Department of Clinical Oncology, Queen Elizabeth Hospital, Kowloon, Hong Kong

## Abstract

Plants including *Rhizoma polgonati*, *Smilax china*, and *Trigonella foenum-graecum* contain a lot of diosgenin, a steroidal sapogenin. This bioactive phytochemical has shown high potential and interest in the treatment of various disorders such as cancer, diabetes, arthritis, asthma, and cardiovascular disease, in addition to being an important starting material for the preparation of several steroidal drugs in the pharmaceutical industry. This review aims to provide an overview of the in vitro, in vivo, and clinical studies reporting the diosgenin's pharmacological effects and to discuss the safety issues. Preclinical studies have shown promising effects on cancer, neuroprotection, atherosclerosis, asthma, bone health, and other pathologies. Clinical investigations have demonstrated diosgenin's nontoxic nature and promising benefits on cognitive function and menopause. However, further well-designed clinical trials are needed to address the other effects seen in preclinical studies, as well as a better knowledge of the diosgenin's safety profile.

## 1. Introduction

Currently, innovative approaches are being adopted to combat the onset and progression of human health problems [1–3]. One strategy involves using compounds derived from edible dietary plants, seeds and traditional medicinal herbs that have an impact on not only treating but also preventing cancer, either alone or along with existing treatment approaches [4–6]. Natural medicines which are derived from medicinal herbs or different vegetables or plants and fruits with promising health benefits are proved to be effective in treating and also preventing a wide range of human ailments, including obesity, neurological disorders, cancer, metabolic syndromes, cardiovascular disease, and diabetes [7–10]. A recent review by Newman and Cragg [11] reported the various natural products as a source of safe drugs for the treatment of various ailments in humans [11]. Over the decades, natural compounds derived from Chinese herbs have been used to a large extent in traditional practices of medicine. There are natural compounds that can be taken from plants, such as the anticancer medicine paclitaxel from *Taxus brevifolia* quercetin found in numerous vegetables and fruits [12, 13] and the antimalarial medication artemisinin from *Artemisia apiacea*, which have a wide spectrum of pharmacological effects [14].

Sapogenins are a group of chemicals that are found in a variety of natural products in glycoside form and enhance overall health. The most effective bioactive chemicals derived from natural product sources are steroidal sapogenins (otherwise known as spirostans) [15, 16]. The majority of steroidal sapogenins show pharmacological action in vitro and in preclinical animal models [15, 17]. Sapogenins have been the subject of several clinical investigations, which are either finished or ongoing [18, 19]. Diosgenin is a steroidal sapogenin that can be found in the *Rhamnaceae*, *Liliaceae*, *Scrophulariaceae*, *Dioscoreaceae*, *Amaryllidaceae*, *Solanaceae*, *Leguminosae*, and *Agavaceae* families [20–23]. It is plentiful in *Rhizoma polgonati*, *Smilax china, Dioscorea villosa*, *Trigonella foenum-graecum*, and Dioscorea rhizome, among other plants with medicinal values. In the pharmaceutical sector, diosgenin is considered to be a fundamental material for starting the steroidal medicines to synthesize. One of the leading causes of death in the whole world is considered to be chronic diseases, and despite recent advancements in the procedures of treatment, synthetic medications still exhibit grating side effects along with chemoresistance, restricting the usage of these medications.

Phytochemicals have gained popularity due to their low risk of negative effects [8, 24]. Diosgenin is a phytochemical that has acquired prominence due to its usefulness in the treatment of lethal diseases such as nervous system disorders, diabetes, cancer, arthritis, asthma, cardiovascular disease, and others [25]. Scientists have used it in the treatment of inflammation, malignancies, hyperlipidemia, and infections as it has a wide spectrum of therapeutic attributes and pharmacological actions [26]. Diosgenin has been investigated extensively for the management and treatment of cancer [17], skin illnesses [27], cardiovascular illnesses [28], diabetes mellitus [29], atherosclerosis [30], and osteoporosis [31].

Diosgenin has been largely talked of and written about for its huge potential in pharmacological studies, as well as the intriguing rudimentary means of action, validating and expanding the comprehensive knowledge gained through its conventional use. Several mechanistic and preclinical studies have been conducted in this context, primarily during the last two decades, to better apprehend the true advantages and significance of diosgenin against manifold illnesses [32, 33]. Overall, the findings of multiple investigations suggest that diosgenin could be used as a novel multi-target-based therapeutic or chemopreventive drug for a variety of chronic diseases. As a result, it has become an agenda of great interest to develop effective ways to derive diosgenin from various natural resources, furthermore to establish medication dosage forms to grant its administration [34, 35]. Recently, the role of diosgenin in diabetic and cardiac diseases has been reviewed, but a holistic review of various biological activities based on preclinical and clinical studies is still missing in the literature to the best of our literature review [36, 37]. The fundamental objective of this review is to summarize the in-depth pharmacological activity moving from preclinical to clinical evidence and safety issues, to upcoming strategies to overcome present limitations.

## 2. Review Methodology

To conduct this comprehensive study, the most relevant studies on the pharmacological properties of diosgenin were analyzed. We searched for scientific publications published in journals electronic databases such as PubMed/MEDLINE, DOAJ, Scopus, Web of Science, and SciFinder, using the next MeSH terms: “Diosgenin/analogs & derivatives,” “Diosgenin/pharmacology,” “Diosgenin/therapeutic use,” “Humans,” Anti-Asthmatic Agents/pharmacology” “Chronic Disease/prevention & control,” “Cardiovascular Diseases/drug therapy,” “Inflammation/drug therapy,” “Neoplasms/drug therapy,” “Nervous System Diseases/drug therapy” “Saponins/pharmacology,” “Saponins/therapeutic use,” “Saponins/toxicity,” “steroids,” and “Signal Transduction/drug effects.” Studies that explained the molecular mechanisms, signaling pathways, molecular targets, and well-defined doses of pharmacological experiments were included. Duplicates, experimental pharmacological studies involving other test substances, and homeopathic preparations were excluded. The most relevant information was summarized in tables and images. The taxonomy of plants has been validated according to The Plant List [38, 39] and the chemical formulas verified with the PubChem database [40].

## 3. Sources

The compound diosgenin was discovered by Fuji and Matsukawa in 1936 [41], and its application in the synthesis of cortisone and other drugs was reported by Marker and coworkers [42]. The *Dioscorea* genus is the main source of diosgenin, and ~137 species of this genus contain diosgenin [26] including *Dioscorea nipponica* [43], *Dioscorea zingiberensis* [44], *Dioscorea composita* [45], and *Dioscorea deltoidea* [46, 47]. It is also isolated from other botanicals including *Trigonella foenum-graecum*, *Costus speciosus*, *Tribulus terrestris* L., *Rhizoma polgonati*, and *Paris polyphylla* (Table 1). The compound diosgenin is mostly produced from the hydrolysis process of steroidal saponins in the presence of strong acid, base, or enzyme catalyst, while microbial transformation technics are gaining much attention due to their highly specific nature, low cost, and environmentally friendly conditions [48].

## 4. Biosynthesis

**Figure 1:**
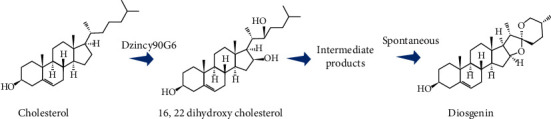
The biosynthetic pathways of diosgenin. *Dioscorea zingiberensis*, for diosgenin biosynthesis uses two P450 genes, DzinCYP94D144 and DzinCYP90G6, the orthologs of PpCYP94D108 and PpCYP90G4, which, from cholesterol, catalyze the formation of diosgenin.

Microwave-assisted extraction (MAE) and ultrasound-assisted extraction (UAE) methods were used for a better yield of diosgenin by using different solvent systems including acetone, ethanol, hexane, and petroleum ether with different concentrations (40, 60, 80, and 100%) and treatment time (MAE: 1.5, 3.0, 4.5, and 6.0 min; UAE: 30, 40, 50, and 60 min). The result of this study indicates that different parameters such as solvent type, concentration, treatment time, and extraction method have a significant impact on diosgenin extraction (yield). The UAE method (21.48%, 40.37 mg/100 g) showed better yield and diosgenin content compared to the MAE method (7.38%, 35.50 mg/100 g) at 80% ethanol concentration at 6 and 60 min, respectively [49]. In the industry of pharmaceutical sector, most hormonal medicines have diosgenin as their precursor. This makes diosgenin a classic precursor which is generated mostly by *Dioscorea* species. The mechanisms underlying the emergence and evolution or progression of biosynthesis of diosgenin in plants are unknown. The geneses of the diosgenin biosynthesis pathway, as well as its evolution, in yam, were validated in recent experimental research. The authors used a variety of diosgenin biosynthesis routes in this study [55] (Figure 1).

Metabolic engineering has also been used to produce diosgenin from yeast. Because cholesterol biosynthesis is more efficient in animal cells than in plant cells, researchers have created a strain with yeast chassis that produces cholesterol by integrating animal genes [56]. As a common precursor for triterpenoid biosynthesis, 2,3-oxidosqualene could be produced in significant amounts from it; *Saccharomyces cerevisiae* strain BY-T3 which was previously modified was chosen as the starting strain [57]. First, Cheng and coauthors discovered that positive selection-driven P450 gene neofunctionalization and duplication were significant in the diosgenin biosynthesis pathway formation. They discovered that CpG islands, which developed to maintain the balance of carbon flux between the production of diosgenin and starch and modulate or control gene expression in the diosgenin pathway, were responsible for diosgenin enrichment in the yam lineage. Finally, we heterologously produced diosgenin to 10 mg/L in yeast that was genetically modified by combining genes from plants, mammals, and yeast [55].

## 5. Pharmacological Properties of Diosgenin: Underlying Molecular Mechanisms and Signaling Pathways

### 5.1. Neuroprotective

The oxidative stress caused by the reactive oxygen stress leads to various ailments such as neurological disorders, cardiovascular diseases, and cancer [58–60]. Health benefits of diosgenin administration are most commonly determined as a neuroprotective agent in terms of cognitive effects [61–63], neuroinflammation [51, 64–67], multiple sclerosis [68, 69], spinal cord injury [70, 71], stroke and thrombosis [72], and neuropathic pain [43, 73–75]. Few recent investigations based on diosgenin and related aspects have been studied through in silico, in vitro, and in vivo approaches and discussed here. Regarding this, a new structure of the neuronal network has been developed to calculate the solubility of the diosgenin compound. The ordinary neural network modeling was used to improve the calculation accuracy and estimate the solubility of diosgenin in the n-alkanols with more carbon atoms due to its rational design concept [76].

In another study, diosgenin was evaluated against cerebral ischemia-reperfusion injury through in silico (proteome dynamic approach), in vitro, and in vivo methods. The authors investigated 5043 regulatory proteins from the brain samples and different signaling pathways [77]. Screening of small molecules for targeting the fungal virulence factors without any effect on viability was conducted by Aaron and the group [78]. They demonstrated that *Cryptococcus neoformans* (*Cn*) was prohibited from crossing the blood-brain barrier via suppressing the proteolytic activity of Mpr 1 with maintaining *Cn* viability. In another word, out of 240 compounds, diosgenin with two other compounds significantly inhibited Mpr 1 proteolytic activity (IC_50_: <10 *μ*M) without any cell toxicity and blocked *Cn* crossing the BBB [78].

Cheng et al. evaluated the antiapoptotic effects of diosgenin in D-galactose-induced ageing brain (cerebral cortical apoptosis) [79]. A total of 36 male (12-week-old) Wistar rats were recruited and divided into four groups (control: 1 mg/kg/day of saline, i.p.; DD0: 150 mg/kg/day of D-galactose, i.p.; DD10: D-galactose +10 of diosgenin; and DD50: D-galactose+50 mg/kg/day of diosgenin orally) and treated up to 8 weeks. The overall results of this study showed the suppressing (D-galactose-induced neuronal Fas-dependent and mitochondria-dependent apoptotic pathways) and enhancing (Bcl-2 family-associated prosurvival and IGF-1-PI3K-AKT survival pathways) effects of diosgenin, which may trigger neuroprotective effects against D-galactose-induced ageing brain [79].

The neuroprotective effects of diosgenin in the diabetic mice model were also evaluated by Leng and the group [80]. All mice (male C57) were fed for up to 8 weeks (high fat diet) and intraperitoneally injected with streptozotocin (dose: 100 mg/kg for 2 days). Eligible mice were dived into four groups including control (*n* = 6), diabetic group (*n* = 6), low-dose diosgenin group (*n* = 6, 50 mg/kg), and high-dose diosgenin group (*n* = 6, 100 mg/kg). The main outcome of this study indicates that diosgenin significantly reduced the level of blood glucose and increased the body weight of diabetic mice. Diosgenin attenuated the level of MDA (in dose-dependent manner) but increased the activity of antioxidant enzymes (superoxide dismutase (SOD) and glutathione peroxidase (GPx)) and expression of signaling pathways (nuclear factor-erythroid factor 2-related factor (Nrf2), heme oxygenase (HO), and NAD(P)H dehydrogenase [quinone] (NQO)-1) in diabetic mice. These signaling pathways were involved in its neuroprotective activities [80].

Oyelaja-Akinsipo et al. investigated the neuroprotective and glucose-lowering ability of compound diosgenin in hyperglycemia-mediated cerebral ischemic brain injury using the zebra-fish model of type II diabetes mellitus [81]. Diabetes was developed in an experimental model by using streptozotocin (20 mg/kg b.w.) for 28 days, and two different doses (20 and 40 mg/kg b.w) of diosgenin were used. The results of this study indicated that diosgenin significantly decreased the concentration of glucose from 175.87 to 105.68 mg/d/L and 82.06 mg/d/L in both doses. The compound treatment increased the body weight and growth in diabetic zebra fishes (*p* < 0.05 and *p* < 0.001) and also enhanced the catalytic activity. Its activity also protects the brain from the possibility of hyperglycemic-mediated brain injury and apoptotic brain cell death [81].

The most representative studies on neuroprotective activities of diosgenin have been presented in Table 2.

### 5.2. Anticancer

Cancer is the most dangerous disease that affects people around the globe, with one in every six deaths due to cancer [92–94]. Different therapeutic tactics, including radiotherapy, chemotherapy, and laser-based therapy, are currently under practice [59, 95, 96]. A series of experiments on cytotoxicity aspects of diosgenin was conducted by several researchers worldwide. In a recent study, cytotoxic effects of standardized extracts, fraction and their compounds (*Trigonella foenum-graecum*) were evaluated against human cancer cells (SKOV-3, HeLa, and MOLT-4 cells). The steroid saponins fraction (C) showed strongest cytotoxic activity on cancer cells (IC_50_: 3.94 (HaCaT), 3.91 (HeLa), 3.97 (SKOV-3), and 7.75 (MOLT-4)). The fraction significantly increased reactive oxygen species production and caspases activity in the cells [97].

A total of 28 diosgenin amino acid ester derivatives (3a-3g and 7a-7g) were designed and synthesized by Ma et al. and evaluated for their cytotoxicity against six human cancer cells including K562, T24, MNK45, HepG2, A549, and MCF-7 [98]. The majority of derivatives displayed cytotoxic potential against these six tumor cells. Out of 28 derivatives, compound 7g exhibited significant cytotoxicity against the K562 cells (IC_50_: 4.41 *μ*M) compared to diosgenin (IC_50_: 30.04 *μ*M). Compound 7 also triggered K562 cells apoptosis through mitochondria-related pathways. The cytotoxicity of two different extracts of *Paris polyphylla* rhizomes (ethanol extract and diosgenin rich extract) were evaluated against human breast cancer cells (MCF-7 and MDA-MB-231), cervical cancer cells (HeLa), and Hep-2 cell lines. The diosgenin-rich extract significantly reduced the proliferation of all cancerous cells, and the maximum activity was observed in MCF-7 cells. Diosgenin-rich extract triggered upregulation of Bax and downregulation of Bcl-2 and BIRC5 pre-mRNA transcripts of genes [54].

Yin and coworkers developed and synthesized thirty-two new diosgenin derivatives and evaluated their cytotoxic activity against three human cancer cells (A549, MCF-7, and HepG2). Among them, compounds 8, 18, 26, and 30 were more potent compared to diosgenin. Compound 8 showed strong and low cytotoxic activity against HepG2 cells (IC_50_: 1.9 *μ*M) and low L02 cells (IC_50_: 18.6 *μ*M), respectively. Additionally, compound 8 induces *G*_0_/*G*_1_ cell cycle arrest and apoptosis in HepG2 cells. A molecular docking study also suggested that p38*α*-MAPK is a suitable target for compound 8 and fits well its active site [99]. Two new azasteroids were synthesized from diosgenin through the modification in the A and B rings and evaluated for their antiproliferative activity [100].

**Figure 2:**
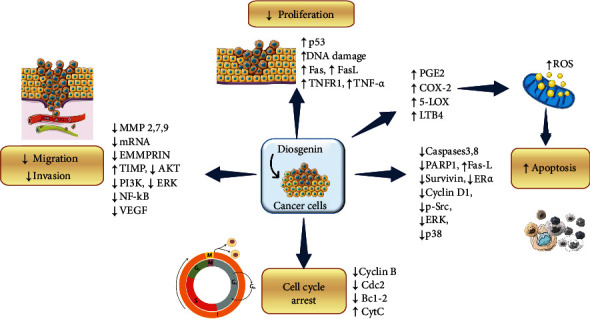
Diagram with cellular targets and molecular mechanisms involved in anticancer effect of diosgenin. Abbreviations and symbols: ↑: increased; ↓: decreased; MMPs: matrix metalloproteinases; abbreviated mRNA: messenger RNA; TIMP: metallopeptidase inhibitor 1; AKT: serine/threonine kinase 1; PI3K: phosphatidylinositol-3-kinase; ERK: extracellular signal-regulated kinase; NF-*κ*B: nuclear factor kappa-light-chain-enhancer of activated B cells; VEGF: vascular endothelial growth factor; DNA: deoxyribonucleic acid; TNFR1: tumor necrosis factor receptor 1; TNF*α:* tumor necrosis factor; PGE2: prostaglandin E_2_; COX: cyclooxygenase; LOX: 5-lipoxygenase; LTB4: leukotriene B4; PARP-1: poly(ADP-ribose) polymerase 1; ER*α*: estrogen receptor alpha; Src: proto-oncogene tyrosine-protein kinase; Cyt C: cytochrome C.

Diosgenin significantly inhibited the cell viability and motility of breast cancer cells and stimulate apoptosis via suppression of S-phase kinase-associated protein Skp-2 in breast cancer cells [101]. Although NF-*κ*B promotes cancer initiation and development, but some reports demonstrate its role in tumor suppression [102]. Part of this several studies has been conducted to investigate the role of diosgenin, and its derivatives against different types of cancers are presented in Figure 2 and Table 3.

### 5.3. Antiatherosclerosis

Atherosclerosis is a disease of the middle and large arteries characterized by the formation in the inner tunic and middle deposits of atheromatous plaques, which contain accumulations of LDL-cholesterol, lipophage, and sometimes calcifications on former lesions that prevent normal blood flow through the vessel. It is a disease in which plaque accumulates inside the arteries and can trigger serious problems including heart failure, stroke, or death [132–134]. The pathogenic mechanisms are complex, involving lipid peroxidation, oxidative stress, inflammation, or altered immune response, causing aging and degenerative brain damage [135–137].

**Figure 3:**
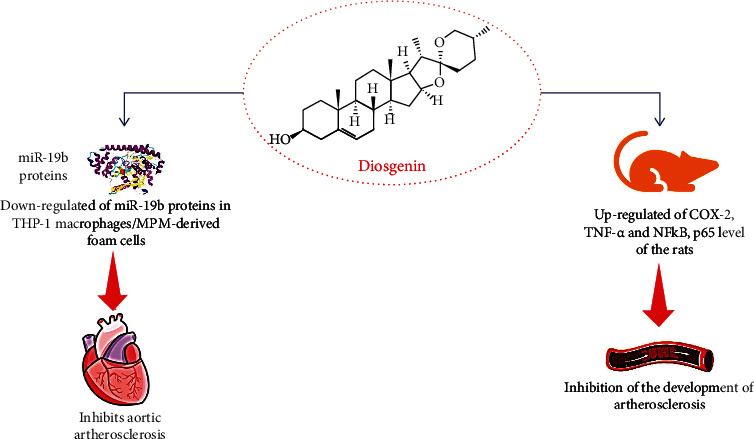
Schematic diagram showing the beneficial effect in atherosclerosis, thus preventing cardiovascular and neurodegenerative diseases. Abbreviations: Cox-2: cyclooxygenase-2; TNF-*α*: tumor necrosis factor-alpha; NF-*κ*B: nuclear factor kappa-light-chain-enhancer of activated B cells.

The antiatherosclerosis potential of diosgenin and its derivatives were studied by few researchers. In this context, the curative effects of diosgenin on macrophage cholesterol metabolism and its mechanism were investigated by Lv and the group [30]. The diosgenin treatment significantly enhanced the expression of ATP-binding cassette transporter A1 (ABCA1) protein without any effect on liver X receptor *α* levels. Additionally, diosgenin treatment also inhibits aortic atherosclerosis progression via downregulation of miR-19b proteins in THP-1 macrophages/MPM-derived foam cells (Figure 3). The in vivo study of the diosgenin compound and its impact on Wistar rats treated with an atherogenic diet were performed by Binesh and coworkers. In this study, the atherogenic diet triggered the inflammatory mediators in the heart, liver, and brain via upregulation of COX-2, TNF-*α*, and NFkBp65 levels of the rats, while diosgenin treatment downregulated the level of these inflammatory markers and inhibit the development of atherosclerosis (Figure 3) [64]. The same research group reported the downregulation of NF-*κ*B expression and polarization of macrophages by diosgenin treatment [138]. In another study, compound dioscin was evaluated for its inhibitory activity against atherosclerosis and postmenopausal atherosclerosis in ovariectomized LDLR-/-mice [139, 140].

### 5.4. Antiasthmatic

Asthma is a long-term condition which results in inflammation of the lower respiratory tract and affects children and adults. Junchao and coworkers evaluated the molecular mechanism of anti-trachea inflammatory effects produced by diosgenin via interactions with glucocorticoid receptor alpha [141]. They used ovalbumin-induced asthmatic mice and primary tracheal epithelial cells as experimental models. The results demonstrated that diosgenin significantly reduces the secretion of different inflammatory factors including tumor necrosis factor (TNF)-*α*, interleukin (IL)-1*β*, and IL-6 via upregulation of glucocorticoid receptors, secretory leukocyte protease inhibitor, glucocorticoid-induced leucine zipper, mitogen-activated protein kinases (MAPK) phosphatase 1, and downregulation of heat shock proteins (HSP70).

### 5.5. Hepatoprotective

**Figure 4:**
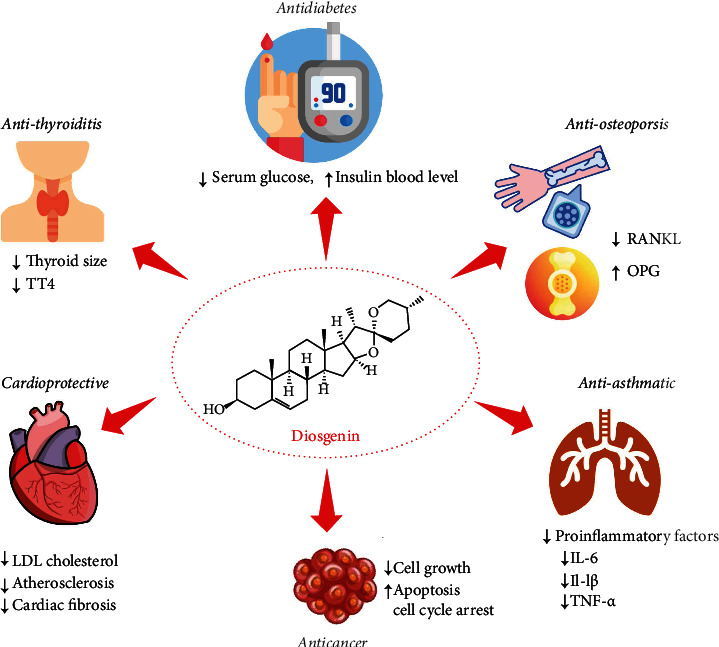
Summarized scheme showing the most representative biological activities of the diosgenin. Abbreviations and symbols: ↑: increase; ↓: decrease; T4: thyroxine; LDL: low-density lipoprotein; OPG: osteoprotegerin; RANKL: receptor activator of nuclear factor kappa beta; IL: interleukin; TNF-*α*: tumor necrosis factor-alpha.

Liver disease can have genetic causes or it can be caused by a variety of factors that affect the liver, such as viruses or alcohol consumption [142–144]. Obesity is also associated with liver disease. Over time, liver damage results in scarring (cirrhosis), which can lead to liver failure, a life-threatening condition [145, 146]. Under this topic, Xie et al. [147] reported on the impact of diosgenin on transforming growth factor (TGF)-*β*1-induced hepatic stellate cells and its mechanism of action for antifibrotic effects. The results of this study showed that diosgenin significantly inhibited the proliferation of TGF-*β*1-induced hepatic stellate cells and reduced the expression of collagen I and alpha-smooth muscle action as well as the expression of TGF-*β* receptors I and II. Additionally, diosgenin also downregulated the expression of TGF-*β*1-induced phosphorylation of Smad3 in hepatic stellate cells and displayed potential effects to treat liver fibrosis. In another study, Zhang and coworkers described the dioscin and its effect on alcoholic liver fibrosis (in vitro and in vivo). The results demonstrated that dioscin improved the condition of alcoholic liver fibrosis via modulation of toll-like receptor 4/myeloid differentiation primary response 88/NF-*κ*B signaling pathway [148]. Other important studies related to diosgenin and its derivatives have been presented in Table 4 and shown in Figure 4.

## 6. Clinical Studies

A clinical pilot study was conducted to investigate the efficacy and safety profile of diallyl thiosulfinate associated with nuciferine and diosgenin in the cure of primary and secondary erectile dysfunction. A total of 143 candidates (age 18-39 Y) were selected and treated orally with nuciferine and diosgenin for up to three months (single dose, alternative days). After three months of treatment, each candidate was screened in terms of the international index of erectile-5, premature ejaculation diagnostic tool, and male sexual health questionnaire. A significant enhancement was observed in terms of the international index of erectile-5 (8.7 vs. 14.01; *p* < 0.001) when compared with baseline and follow-up visits. The overall observation of the study indicates that these three compounds are capable to enhance the control of ejaculation in candidates suffering from premature and erectile dysfunction without any side effects [157].

In another study, Tohda et al. evaluated the impact of diosgenin-rich yam extract on synaptic loss and memory dysfunction using a transgenic mouse model of Alzheimer's. A placebo-controlled, randomized, double-blind, cross-over study was performed on 28 healthy volunteers (age: 20–81 years) and randomly treated with yam extract or placebo (12-weeks intake and 6-week washout period). The Japanese version of the Repeatable Battery for the Assessment of Neuropsychological Status (RBANS) test and the adverse effects were evaluated. The diosgenin-rich yam extract consumption (12 weeks) significantly increased the synaptic fluency or enhance cognitive function without any side effects [158].

The standardized multinutrient supplement including folic acid, selenium, vitamin E, catechins, glycyrrhizin, diosgenin, damiana, and omega-3-fatty acids showed beneficial effects on in vitro fertilization/intracytoplasmic sperm injection in terms of embryo quality [159]. The curative effects of *Dioscorea villosa* extract on menopausal systems, lipids, and sex hormones were evaluated [160].

A placebo-controlled, randomized, double-blind, cross-over study was conducted on 23 healthy women suffering from symptoms of menopause. All candidates were treated with wild yam cream or placebo for up to three months, and no significant adverse effects were observed in both the treatments. Additionally, no changes were also recorded in weight, systolic or diastolic blood pressure, total serum cholesterol, triglyceride, high-density lipoprotein cholesterol, glucose, estradiol, or serum [160].

## 7. Toxicological Profile: Safety and Side Effects

Only a few articles have acknowledged diosgenin's toxicity, even though there has been substantial research on its part and maneuver in the treatment along with prevention of cancer and further different chronic disorders. A study was published in 2009 on breast cancer cells by Srinivasan and coworkers where it was shown that diosgenin modulates AKT to regulate breast cancer cell survival and that this drug has no effect on normal breast epithelial cells (MCF-10A) except for its selective toxicity to cancer cells [108].

Steroidal saponins of different doses from *D. zingiberensis* were given to mice that were experimental in another in vitro investigation. Diosgenin was the metabolite and the main component of these saponins. There were no harmful effects up to a level of 562.5 mg/kg, according to the findings. However, steroidal saponins, which include diosgenin, displayed deleterious consequences and even death in a dose-dependent manner at dosages of 1125 mg/kg and higher. Interestingly, the steroidal saponins dosage, which is traditional, is 510 mg/kg/day, implying that steroidal saponins, together with diosgenin, have no significant toxicity at this dosage [161]. A recent study also discovered that diosgenin derivatives had antithrombotic properties. In vivo experiments revealed that they appeared to be protective and comparable to aspirin, with a decreased risk of bleeding and less stomach mucosal injury [162]. Furthermore, investigations have shown that diosgenin has a modest inhibitory impact on cytochrome P450 enzymes (CYPs), suggesting diosgenin fused with any other medicine would be safe to consider that they would have no toxicity [163]. These investigations demonstrated that diosgenin and its derivatives are nontoxic and have underlined their utility in the medicaments of chronic disorders including cancer.

## 8. Therapeutic Perspectives and Limitations

Diosgenin is a natural compound mostly found in *Dioscorea* species and starting material for the commercial synthesis of different steroids including cortisone, pregnenolone, and progesterone. The natural antioxidant compound diosgenin possesses different biological activities such as anticancer [164, 165], antidiabetic [28, 36, 152, 166–169], multiple sclerosis [68, 69], and spinal cord injury [70, 71] and helps in the management of these diseases. Therefore, diosgenin could be functional and helpful in the treatment and prevention of a variety of disorders. The identification of diosgenin-specific targets, on the other hand, is critical for further validating its use in the treatment and elimination of diseases.

The main therapeutic limitations of diosgenin are represented by its low bioavailability; therefore, a special emphasis is being paid to the production of nanoformulations or conjugate complexes to improve the compound's bioavailability and pharmacokinetic features to develop it into a possible medicine. The potential of this chemical, its analogs, or combinations of this molecule with others has previously been demonstrated; however, carrier systems such as nanoparticles must be developed to govern diosgenin to the location where it works, boosting effectiveness and lowering adverse impacts. For example, encapsulated diosgenin PCL [poly(caprolactone)]-pluronic nanoparticles (PCL-F68-D-NPs) were developed by the nanoprecipitation method to improve performance in brain cancer therapy [170]. The developed nanoparticles (PCL-F68-D-NPs) displayed significant cytotoxicity against U87-MG cells compared to free diosgenin. Additionally, developed nanoparticles demonstrated suitable properties in terms of size distribution, stability, morphology, chemical and mechanical properties, encapsulation, and loading efficiency [170].

## 9. Overall Conclusions

A steroidal saponin, diosgenin, and its chemical and structural variants are useful in the treatment of a great variety of chronic conditions, including cardiovascular disease, several forms of lethal malignancies, nervous system problems, and autoimmune diseases. They have gotten a lot of interest from researchers all across the world. Diosgenin and its derivatives have been shown to have pharmacological benefits against cancer, diabetes, osteoporosis, Alzheimer's disease, and stroke in several investigations. Diosgenin has been shown to act on several molecular targets that are essential players in the occurrence and incidence of many serious disorders. Its multitargeting capability allows it to influence multiple molecular targets and signaling pathways at the same time. Diosgenin has an edge over the most commercial medicinal medicines available today because of this property. Furthermore, investigations demonstrating its nontoxic nature significantly promote the inclusion of this medicine in additional clinical studies or trials in the forthcoming days. Overall, diosgenin demonstrated great promise in the treatment and prevention of a variety of chronic diseases; nevertheless, additional clinical research is needed in the nearing days to come to confirm the preclinical findings and demonstrate the effective and secure usage of these purely natural compounds.

## Figures and Tables

**Table 1:** Botanical sources of diosgenin.

SN	Botanical name	Family	References
1	*Trigonella foenum-graecum*	Fabaceae	Arya and Kumar [49]
2	*Costus speciosus*	Costaceae	Selim and Al Jaouni [50]
3	*Tribulus terrestris* L	Zygophyllaceae	Wang et al., [51]
4	*Smilax china* L.	Smilacaceae	Yin et al. [52]
5	*Rhizoma polgonation*	Asparagaceae	Chen et al. [53]
6	*Helicteres isora* L.	Malvaceae	Deshpande and Bhalsing [21]
7	*Paris polyphylla*	Melanthiaceae	Gupta et al. [54]

**Table 2:** Pharmacological studies regarding diosgenin's neuroprotective effect with mechanism of action.

Tested compounds	Model	Potential mechanisms	References
Diosgenin	C6 rat glioma cells in vitro	↓ the dosage regimen of TMZ, ↑ MMP-2, ↑ apoptosis	Rajesh et al. [82]
Diosgenin	5XFAD mice in vivo	↓amyloid plaques, ↓neurofibrillary tangles in the cerebral cortex and hippocampus, ↑1,25D_3_-MARRS	Tohda et al. [83]
Diosgenin	ddY mice in vivo	Memory enhancement effects mediated by 1,25D_3_-MARRS-triggered axonal growth, ↑ 1,25D_3_-MARRS	Tohda et al. [84]
Diosgenin	Transgenic 2576 mice in vivo	↓AChE, ↓Bax/Bcl-2, ↓amyloid plaques production in the granule cells, ↑ NGF, ↑ SOD, p^75^ (NTR)	Koh et al. [85]
Diosgenin	Senescent mice induced via D-galactose in vivo	↑learning and memory ability, ↑ SOD, ↑ GSH-Px, ↓ MDA level, ↑ endogenous antioxidant enzymatic activities	Chiu et al. [61]
Diosgenin-rich extract	Senescent mice induced via D-galactose in vivo	↑learning and memory ability, ↑ SOD, ↑ GSH-Px, ↓ MDA level, ↑ endogenous antioxidant enzymatic activities	Chiu et al. [86]
Diosgenin	Sprague-Dawley rats in vivo	↓ neuronal death rate, ↓pro-inflammatory cytokines, ↑impaired neurological functions at 100 and 200 mg/kg, ↑ IkB*α*, ↓ p65, ↓NF-*κ*B	Zhang et al. [87]
Diosgenin + curcumin (bivalent)	MC65 neuroblastoma cells in vitro	Antioxidant, anti-oligomerization ↓ amyloid-*β* oligomer formation	Chojnacki et al. [88]
Diosgenin	Sprague-Dawley rats with lipopolysaccharide in vivo	↓TLR/NF-*κ*B ↓ TLR2, ↓TLR4, ↓NF-*κ*B	Li et al. [89]
Diosgenin carbamate derivatives	D-galactose aging mice in vivo	Anti-inflammatory, anti-oxidant, *β*- amyloid, ↓ NO ↓ IL-1*β*, IL-6, TNF-*α*	Yang et al. [90]
Arginyl–diosgenin	Neuroinflammation model using BV2 cells induced by LPS C57BL/6 mice in vitro/in vivo	↓activation of microglia, microphages, ↓CD4^+^ T cell proliferation, ↓Th1/Th17 cell differentiation, ↓ NO, ↓ iNOS, ↓ COX-2, ↓PGD2, ↓IL-6, ↓IL-1*β*, ↓ TNF-*α*	Cai et al. [65]
Diosgenin	Primary murine microglial cell line BV-2 in vitro	↓pro-inflammatory M1 markers via activation of microglia and without affecting M2 makers, ↓I*κ*B-*α*, ↓ERK, ↓ MAPK, ↓p38	Wang et al. [51]
Diosgenin	SH-SY5Y cell line H9c2 cell line in vitro	Protective effects against SH-SY5Y cells, ↓angiogenesis at high concentration, ↓apoptosis	Cai et al. [91]
Diosgenin	Trimethyltin-injected transgenic 2576 mice in vivo	Neuroprotective effects against different brain damages via NGF biosynthesis stimulation, ↓ AChE, ↓ Bax/Bcl-2, ↑ NGF, ↑ SOD	Koh et al. [85]

Symbols: ↑ = increased; ↓ = decreased.

**Table 3:** Anticancer properties of diosgenin and their derivatives in different types of cancer.

Model/cancer cell lines/IC_50_	Mechanism	Pharmacological action	References
MCF-7 Hs578T IC_50_ = 0–40 *μ*M	G2/M phase arrest, ↓cyclin B, ↓ Cdc2, ↓ Bcl-2 ↑caspase 3	Regulation of the level of proteins which triggered cell cycle blockade at the G2/M phase	Liao et al. [103]
MDA-MB-231 IC_50_ = 5 *μ*M	**↓** Cdc42 **↓** Vav2	↓ cancer cell migration ↓actin polymerization ↓Vav2 phosphorylation ↓Cdc42 activation	He et al. [104]
MCF-7 IC_50_ = 10 − 30 *μ*M	**↓**caspases-3,8, ↓PARP1, ↑Fas-L, ↓Survivin, ↓ER*α*, ↓cyclin D1, ↓c-Myc, ↓p-Src, ↓ ERK½, ↓p38	↓expression of ER-*α* ↑apoptosis via extrinsic pathway	Chun et al. [105]
MCF-7 MDA-MB-231 IC_50_ = 1.15 − 5.76 *μ*M	**↑** GATA3, **↑** DNMT3A, **↑** ZFPM2, **↑** E-cadherin, **↑** TET2, **↑** TET3, **↓** TET1, **↓** vimentin, **↓** MMP9	Diosgenin mediated pathways modulate the GATA3 expression at transcription and translation	Aumsuwan et al. [106]
MDA-MB-231 MDA-MB-453 T47D IC_50_ = 0 − 8 *μ*g/mL	↓ Bcl-2 ↓ cIAP-1 ↓ McI-1	↑ apoptosis via downregulation of proteins related with inhibition ↑apoptotic process	Kim et al. [107]
MCF-7 MCF-10 MDA-231 IC_50_ = 20 − 30 *μ*M	↓ pAKT (Ser473), ↓ AKT kinase activity, ↓ p-GSK3*β*, ↓ Raf, ↓ p-MEKs 1/2, ↓ p-MEKs 3/6, ↓ MEK-1, ↓ MEK-4, ↑ pElk-1, ↑ p21, ↓ XIAP, ↓ Bcl-2, ↓ Cdk-2, ↓ Survivin, ↓ cyclin D1, ↓ NF-*κ*Bp65, ↓ p65, ↑ I*κ*B-*α*, ↓ pElk-1, ↑ Bax, ↓ NF-*κ*B, ↑ caspase-3	↑ G1 cell cycle arrest apoptosis in MCF-7 and MDA-231 cells while did not cause in MCF-10A cells	Srinivasan et al. [108]
HuCCT1 QBC939 HuH28 SK-ChA1 RBE Mz-ChA-1 IC_50_ = 0 − 40 *μ*M	↑Bax/Bcl-2, ↑p21, ↑caspase-3, ↑ pARP-1,↓CyclinB1 ↑Cyt c, ↑GSK3*β*-PY216 ↓ GSK3*β*-PS9	G2/M phase arrest ↑apoptosis The compound suppressed cholangiocarcinoma cells and triggered	Mao et al. [109]
HT-29 HCT-116 IC_50_ = 40 *μ*M	↑ PGE2, ↑ COX-2, ↑ 5-LOX, ↑ LTB4	↑ apoptosis in both cancer cell lines	Lepage et al. [110]
HCT-116	↑ ROS, ↑ Ca2+, ↑ NO, ↑ iNOS, ↑ DNA damage, ↑ Gna11, ↑ATP6V0C, ↑Ppp2r5e, ↑COX6C ↑mRNA	The compound triggered mitochondrial damage and G2/M cell cycle arrest	Chen et al. [111]
HeLa CaSki	↑ caspases-3, 8, 9	Diosgenin and its glycoside derivatives showed strong anticancer activity with low necrotic activity and selective action	Hernández-Vázquez et al. [112]
HeLa SiHa IC_50_ = 1.25 − 5.0 *μ*g/mL	↑ ROS, ↑ Ca^2+^, DNA damage, ↑ Bid, Bcl-2, ↓ Bcl-xL, ↑ caspases-3, 9, ↑ Bax, ↑ Bak, ↑ p53	↑ apoptosis ↓ cell proliferation, ↑DNA damage in both cell lines via modulation of protein level	Zhao et al. [113]
HeLa	↑ apoptosis, ↑ caspase-3 and -9 activity, ↓ Bcl-2	The compound significantly induced apoptosis in a dose and time-dependent manner	Cai et al. [114]
HeLa IC_50_ = 0 − 40 *μ*M	G2/M phase, ↑ apoptosis, ↑ ROS	The compound significantly inhibits cell proliferation, transformed cell morphology, arrests the cell cycle, and regulates apoptosis via death receptor and mitochondrial pathways.	Ma et al. [115]
KYSE510 IC_50_ = 0.5 − 20 *μ*M	G1/S arrest, ↑ apoptosis, ↑ cleaved caspase-9, ↑ Bax, ↑ Cyt c, ↑ ROS, ↓ Bcl-2	Peroxiredoxins 1 and 6 play an important role in compound induced apoptosis	Zhiyu et al. [116]
NOZ SGC996 IC_50_ = 0 − 8 *μ*M	↓ ROS-mediated PI3K/AKT	↑ apoptosis via inhibition of reactive oxygen species-mediated PI3K/AKT signaling	Song et al. [117]
MGC-803 MKN-45 IC_50_ = 1.25 − 5.0 *μ*g/mL	↑ ROS, ↑ Ca^2+^, ↑ RBM-3, ↑ GALR-2, ↓ CliC-3, ↓ Bcl-2, ↑ Bax, ↑ caspase-3, 9,↑ MAPKs, ↓ CAP-1, ↓ Tribbles-2	Anticancer effects against human gastric cancer via inducing cell apoptosis, DNA damage, etc.	Zhao et al. [118]
SGC-7901 IC_50_ = 0.65 − 2.6 *μ*g/mL	↑ Fas, ↑ FasL, ↑ TNFR1, ↑ TNF-*α*, ↓ Bcl-2, ↑ Bax, ↑ Bak, ↓ bid, ↓ Bcl-xL, ↑ p53 mRNA ↑ caspase-3, 8	Anticancer activity	Hu et al. [119]
HGC-27 MGC-803 SGC-7901 IC_50_ = 0 − 60 *μ*mol	↓proliferation ↓ HOTAIR ↓Hox	↓proliferation of gastric cancer cells	Ma et al. [120]
C6 allograft IC_50_ = 1.25 − 5.0 *μ*g/mL	↑ROS, ↑Ca^2+^, ↑MDA, ↑NO, ↑GSSG, ↓GSH, ↓Bcl-2, Bcl-xL, ↑Bak, ↑Bax, ↑caspase-3, 9	Anticancer activity	Lv et al. [121]
HepG2 IC_50_ = 0 − 100 *μ*M	↓TAZ ↓*β*-catenin	↓ cell growth, ↑apoptosis, ↑ apoptosis, ↑G2/M phase arrest	Chen et al. [122]
HepG2 SMMC-7721 IC_50_ = 0 − 100 *μ*M	G2/M phase arrest, ↑ DDX3, ↓mRNA, ↓ cyclin D1, ↑p21, ↑E-cadherin, ↓ Notch-1, ↓*β*-catenin	↓ cell growth ↑apoptosis via upregulation of DDX3	Yu et al. [123]
HepG2 IC_50_ = 0 − 40 *μ*M	↑ caspase-3, 8, 9 ↑ Bax, ↓Bcl-2, ↓Bid, ↑ROS, ↑ASK1	↓ cell growth ↑apoptosis in HepG2 cells via Bcl-2 protein-mediated pathways	Kim et al. [124]
Bel-7402 IC_50_ = 0.25 − 63 *μ*mol/L	↑ TP53, ↑ Bax, ↓ Bcl-2 ↑ caspase-3	↓cell growth ↑apoptosis via modulation of protein expression	Zhang et al. [125]
HepG2 IC_50_ = 0 − 20 *μ*M	↑ apoptosis, G2/M phase arrest, ↓ cyclin B1, ↑ Bax, ↑ Bcl-2	↓cell growth ↑apoptosis via modulation of protein expression	Wang et al. [126]
DU145 IC_50_ = 0.2 − 100 *μ*g/mL	↑ LC3-II, ↑ caspase-9, ↓ PI3K, ↓AKT, ↓mTOR, ↑ Beclin-1, ↓ Bcl-2	↓cell growth ↑apoptosis, ↑autophagy due to inhibition of mitochondrial pathways	Nie et al. [127]
DU145 IC_50_ = 33 ng/mL	↓vimentin, ↓ Mdm2, ↓ c-Met, ↓ ERK ↓AKT, ↓mTOR	↑ apoptosis ↓ HGF induced ↑ Mdm2, ↑vimentin ↓phosphorylation of Akt, mTOR	Chang et al. [128]
PC-3 IC_50_ = 0 − 30 *μ*M	↓ MMP 2,7,9, ↓mRNA, ↓ EMMPRIN, ↑ TIMP, ↓ AKT, ↓ PI3K, ↓ ERK, ↓c-JNK, ↓NF-*κ*B, ↓VEGF	↓ cancer cell growth ↑apoptosis via modulation of signaling pathways	Chen et al. [129]
PC-3 IC_50_ = 0 − 100 *μ*M	G2/M phase arrest, ↓ NEDD4, ↓ p73, ↑ LATS1, ↓ p-AKT, ↓ TAZ	↓cell growth, ↑apoptosis cell cycle arrest	Zhang et al. [130]
PC3 IC_50_ = 250 − 1000 *μ*M	↑ [Ca^2+^]i, ↑ Mn^2+^	Significant anticancer activity	Sun et al. [131]

**Table 4:** Different biological activities of diosgenin and their derivatives with the mechanism of action.

Disease	Experimental model	Mechanism	Pharmacological action	References
Cardiac fibrosis	Rat cardiac fibrosis cells in vitro IC_50_ = 0 − 10 *μ*M	↓ *α*-SMA ↓ TGF-*β*1 ↓ p-Smad3	↓ proliferation of Ang II-induced cardiac fibrosis, ECM synthesis of rat cardiac fibrosis and expression of TGF-beta 1 and Smad3 phosphorylation in cardiac fibrosis cells	Zhou et al., [149]
Pulmonary hypertension	ICR mice in vivo Dose = 0.1 − 10 mg/kg	↓ NF-*κ*B ↓p50/p65 ↓ MAPK/p38 ↓ iNOS	Pretreatment with diosgenin significantly suppresses the LPS-induced NF-*κ*B, MAPK/p38 activation protective effects against acute lung injury or sepsis	Gao et al., [150]
Graves disease	BALB/c mice in vivo Dose = 20 − 100 mg/kg/day	↓ mRNA, ↓IGF-1, ↓NF-*κ*B, ↓ cyclin D1 ↓PCNA	Diosgenin treatment significantly reduces the TT4 level and thyroid size without affecting TRAb in graves' disease mice	Cai et al., [151]
Diabetes	Albino rats in vivo Dose =5-10 mg/kg/day	↓serum glucose, ↓ MDA, ↑GSH, ↑ SOD, ↑ GPx, ↓ protein carbonyl ↑ catalase	↓ blood glucose, ↓LDL cholesterol ↓cardiovascular risk	Kalailingam et al. [28]
	Wistar rats in vivo Dose = 15 − 60 mg/kg/day	↓ serum glucose	Diosgenin significantly reduced the blood glucose, increase the insulin blood level	Saravanan et al. [152]
	Swiss mice in vivo Dose = 1 mg/mL	↓ *α*-amylase ↓ *α*-glucosidase	Diosgenin demonstrated significant antidiabetic activity	Ghosh et al., [153]
Osteoporosis	Ovariectomized rats	↓ RANKL ↑ OPG	Diosgenin demonstrated significant antiosteoporotic activity compared to OVX control	Zhang et al. [154]
Arthritis	C57BL/6 mice in vivo	↓ p-JAK2, ↓ p-STAT3, ↓ SDH, ↓COX, ↓ SOD, ↑ Bax	Diosgenin treatment significantly inhibited the apoptosis and upregulated the mitochondrial oxidative stress capacity of chondrocytes in experimental mice with osteoarthritis	Liu et al. [155]
Cardiotoxicity	Male Balb/c mice in vivo Dose = 130 mg/kg/day	↓TBARS, ↓ ROS, ↓ caspase-3, ↓ NF-*κ*B, ↑ cGMP, ↑ cAMP, ↓ PDE5	Cardioprotective Diosgenin displayed antioxidant, anti-apoptotic, cGMP modulation activities	Chen et al. [156]

## Data Availability

The data used to support the findings of this study are available from the corresponding author upon request.
